# Correcting for misclassification and selection effects in estimating net survival in clinical trials

**DOI:** 10.1186/s12874-019-0747-3

**Published:** 2019-05-16

**Authors:** Juste Aristide Goungounga, Célia Touraine, Nathalie Grafféo, Roch Giorgi, the CENSUR working survival group

**Affiliations:** 1 Aix Marseille Univ, INSERM, IRD, SESSTIM Sciences Economiques & Sociales de la Santé & Traitement de l’Information Médicale, Marseille, France; 2 Unité de Biométrie, Institut du Cancer de Montpellier (ICM), Univ Montpellier, Montpellier, France; 3 INSERM U1153, Centre of Research in Epidemiology and Statistics Sorbonne Paris Cité (CRESS), ECSTRA Team, Hôpital Saint Louis, Paris, France; 4 Université Paris Diderot, Paris, France; 5 Aix Marseille Univ, APHM, INSERM, IRD, SESSTIM (Sciences Economiques & Sociales de la Santé & Traitement de l’Information Médicale), Hop Timone, BioSTIC (Biostatistique et Technologies de l’Information et de la Communication), Marseille, France

**Keywords:** Cause of death, Cancer clinical trials, Life tables, Excess hazard model, Net survival, Selection bias

## Abstract

**Background:**

Net survival, a measure of the survival where the patients would only die from the cancer under study, may be compared between treatment groups using either “cause-specific methods”, when the causes of death are known and accurate, or “population-based methods”, when the causes are missing or inaccurate. The latter methods rely on the assumption that mortality due to other causes than cancer is the same as the expected mortality in the general population with same demographic characteristics derived from population life tables. This assumption may not hold in clinical trials where patients are likely to be quite different from the general population due to some criteria for patient selection.

**Methods:**

In this work, we propose and assess the performance of a new flexible population-based model to estimate long-term net survival in clinical trials and that allows for cause-of-death misclassification and for effects of selection. Comparisons were made with cause-specific and other population-based methods in a simulation study and in an application to prostate cancer clinical trial data.

**Results:**

In estimating net survival, cause-specific methods seemed to introduce important biases associated with the degree of misclassification of cancer deaths. The usual population-based method provides also biased estimates, depending on the strength of the selection effect. Compared to these methods, the new model was able to provide more accurate estimates of net survival in long-term clinical trials.

**Conclusion:**

Finally, the new model paves the way for new methodological developments in the field of net survival methods in multicenter clinical trials.

**Electronic supplementary material:**

The online version of this article (10.1186/s12874-019-0747-3) contains supplementary material, which is available to authorized users.

## Background

Recent advances in treatment have extended the expected survival of cancer patients up to and even beyond ten years after diagnosis [1]. This leads to a non-negligible risk of death due to other causes than the cancer of interest, more particularly for older patients. In this context it is of importance to account for the competing causes of death, and methods have been developed to estimate the specific survival of the cancer of interest while estimating the specific survival function(s) of the other(s) cause(s) of death [2]. In this context, estimation of cancer-specific survival can be interpreted as the survival for cancer patients in presence of other cause(s) of death. Another approach relies on the estimation of net survival, representing the survival that would be observed if the cancer under study were the only cause of death [3]. A main interest of this latter approach, is to be interpreted as the survival from cancer in the absence of other causes of death. [4].

Estimation of net survival is performed using either “cause-specific methods” or “population-based methods”. Cause-specific methods consider as censored all deaths from noncancer-specific causes and estimate net survival with classical estimators such as Kaplan-Meier (KM) estimator or the Cox-model-based estimator [5]. They assume independence between cancer-related and cancer-unrelated mortality and require accurate information on the causes of death. However, cause of death information do include errors and are sources of misclassifications [6, 7]. In several studies, the rate of misclassification was found to be very low (2 to 7%) up to 5 years of follow-up but this rate became much more important (up to 20%) with long-term follow-up [8]. These rate changes reflect the difficulty of identifying the cause of death over long follow-ups [9], and therefore make inappropriate the use of methods requiring knowledge of the cause of death to estimate net survival (the same reasoning applies in the competing risk setting to estimate cause-specific functions). Furthermore, cause-specific methods rely on the assumption of independence between the censoring process and the occurrence of death from the cancer of interest [5, 10]. A classic example of violation of this assumption concerns age because it has an impact on both death from the cancer of interest and death from other causes (called herein “other-cause mortality”), leading to biased estimations of net survival [5]. To limit this particular source of bias, Pohar-Perme et al. [11] proposed an approach based on the inverse probability of censoring weighting that uses the Nelson-Aalen estimator (each individual estimation is weighted by the inverse probability of the expected survival [12]).

One solution to avoid cause-of-death misclassification is to use population-based methods. Indeed, such methods account for the competing causes of death by adjusting for the population survival rate obtained from population life tables. They rely on the assumption that mortality due to other causes than cancer of interest is the same as the expected mortality in the general population with same demographic characteristics derived from general population life tables. The observed hazard (*λ*_*O*_) is then considered as the sum of the hazard due to the cancer of interest, the excess hazard (*λ*_*E*_), plus the hazard derived from the other-cause of death than cancer of interest ( *λ*_*P*_); this latter quantity is not estimated but drawn from the general population life tables. The net survival is then the survival function that derives from the excess hazard function and this relation may be expressed as: ${S}_E(t)=\exp \left[-{\int}_0^t{\lambda}_E(u) du\right]$. It can be estimated non-parametrically (e.g., Pohar-Perme estimator [12] and doubly robust estimator [13]) or parametrically (e.g., Estève et al. [14], Giorgi et al. [15], or Remontet et al. [16] regression models). Simulation studies have shown that Pohar-Perme estimator and regression model adjusted on demographic covariates have good performances in estimating net survival [17]. The main assumption of population-based methods is that other-cause mortality in the studied group is comparable to that of the general population [9, 18]. However, patients included in a cancer trial are rather selected. Therefore, they are neither representative of all patients diagnosed with the same cancer nor representative of the general population (different characteristics and different other-cause mortality), even within the same area of residence, and their other-cause mortality as derived from the general population life tables will be surely biased (underestimated or overestimated). To our knowledge, only Cheuvart and Ryan [19] have proposed a population-based regression model to analyze long-term cancer clinical trial that accounts for this type of bias. They introduced a rescaling parameter that allows the mortality from other causes of the studied group to differ from the mortality of the general population. However, i) the baseline hazard is a piecewise exponential, which is not very flexible and requires potentially a high number of parameters to estimate in case of long-term follow-up; and ii) the approach relies on grouped-data, which is source of loss of information.

The present article proposes a new flexible population-based model to estimate long-term net survival in clinical trials and that allows for cause-of-death misclassification and for effects of selection. Comparisons with other cause-specific and population-based methods are carried out to examine the new-model limits.

The article is organized as follows. The Methods section describes parametric and non-parametric estimators of net survival as well as the new model and the simulation study. The Results section presents the results of simulations performed with different combinations of proportion of misclassification / degree of selection effect. This section also shows an application to data from a clinical trial on prostate cancer patients. The article ends with a discussion of the findings.

## Methods

### Models and estimators of net survival

#### Data settings, assumptions and notations

To estimate net survival, two setting are defined according to cause of death information. When one considers that this information is available for each patient, net survival is estimated in “cause-specific setting”. But if cause of death information is unavailable, or if one wants to get rid of cause of death, net survival is estimated in “population-based setting”. In this context, cause of death information is indirectly obtained by matching the observed data with other-cause hazard drawn from general population life tables. Indeed, the tables contain daily hazard rate ${\lambda}_{P_i}$ for each matched individual *i* from the general population of interest. The main assumption to consider that, is the fact that the cancer part in the whole mortality is negligible. In consequence, the other-cause hazard of the studied sample is equal to that of general population of interest. In these two settings the common assumption is that excess hazard and other-cause hazard are independent.

Furthermore, in the two settings, net survival may be estimated using non-parametric estimators or parametric models; the latter allow estimating and testing effects of covariates on the excess mortality.

Some well-known estimators presented in the next sub-section (Kaplan-Meier, Nelson-Aalen, Cox) were firstly developed in the overall cause of death setting. For simplicity reason, we present their adaptation directly in the cause-specific setting, where the main change concerns the event indicator.

#### Non-parametric estimators of net survival

Here, we present briefly the properties of 1) one cause-specific method, 2) one mixed population-based and cause-specific method, and 3) one population-based method. The first is the popular Kaplan-Meier (KM) estimator in the cause-specific setting with right-censoring of the times to death from non-cancer causes. The second uses an adaptation of the Nelson-Aalen estimator to account for the informative censoring problem and uses other-cause mortality information from population life tables. The third is the Pohar-Perme (PP) estimator, a reliable estimator of net survival in population-based studies.

##### The Kaplan-Meier estimator

Estimation of net survival in the cause-specific setting leads to consider deaths from cancer as events and to right censor deaths from other causes and live patients. The KM estimator of net survival is then:

$$ {\widehat{\mathrm{S}}}_{KM_E}(t)=\prod \limits_{t_i\le t}\left[1-{\int}_0^t\frac{d{N}_E(u)}{Y(u)}\right] $$

In this equation, *n* is the number of patients, ${N}_E(t)=\sum \limits_{i=1}^n{N}_{E,i}(t)$ is the number of cancer-related deaths up to the time *t* obtained by summing up the individual counting processes *N*_*E*, *i*_(*t*), and $Y(t)=\sum \limits_{i=1}^n\mathbb{l}\left[{t}_i\ge t\right]$ is the at-risk process just before time *t* (i.e., alive or not censored patients; the at risk process counts the subjects who did not experience the event by time *t* and, thus, who are still “at risk” of experiencing the event).

##### A Weighted Nelson-Aalen estimator

In the cause-specific setting, Pohar-Perme et al. proposed an adaptation of the Nelson-Aalen estimator [12]. When the assumption of independence between the censoring process and the cancer death process is violated, mainly due to age, the censoring becomes informative. Using the inverse probability of censoring weighting approach on the Nelson-Aalen estimator [12], Pohar-Perme et al. derived an asymptotically unbiased estimator of the net survival:

$$ {\widehat{\mathrm{S}}}_{wNA_E}(t)=\mathit{\exp}\left(-{\int}_0^t\frac{d{N}_E^w(u)}{Y^w(u)}\right) $$

In this equation, ${N}_E^w(t)={\sum}_{i=1}^n{N}_{E,i}^w(t)$ and ${Y}^w(t)={\sum}_{i=1}^n{Y}_i^w(t)$ are, respectively, the weighted aggregated counting process and the at-risk process. More precisely, ${dN}_{E,i}^w(t)=\frac{dN_{E,i}(t)}{S_{P,i}\left({t}_i-\right)}$ and ${Y}_i^w(t)=\frac{Y_i(t)}{S_{P,i}\left(t-\right)}$, where each of components are weighted, respectively, by *S*_*P*, *i*_(*t*_*i*_−) and *S*_*P*, *i*_(*t*−) the inverse of the individual expected survival derived from population life tables obtained respectively at times *t*_*i*_− and *t*−. Thereafter, we called this estimator, with these types of weights, the weighted Nelson-Aalen (wNA) estimator.

##### The Pohar-Perme estimator

The PP estimator [12] is a reliable non-parametric estimator of net survival developed to overcome some assumptions of excess hazard modeling. It corresponds to the difference between the Nelson-Aalen estimate and the cumulative population of the patients still at risk at each death, where the at-risk process and the counting process are weighted to give greater weight to subjects with high risk of other-cause mortality. The PP estimator of net survival is:

$$ {\widehat{S}}_{PP_E}(t)=\mathit{\exp}\left(-\left[{\int}_0^t\frac{d{N}^w(u)}{Y^w(u)}-{\int}_0^t\frac{\sum \limits_{i=1}^n{Y}_i^w(u){\uplambda}_{P_i}(u) du}{Y^w(u)}\right]\right) $$

where ${N}^w\left(\mathrm{t}\right)=\sum {N}_i^w(t)$ is the sum of the individual all-cause counting process ${N}_i^w(t)$ and with $d{N}_i^w(t)=\frac{d{N}_i(t)}{S_{pi}(t)}$, which, as ${Y}_i^w(t)$, is weighted by the inverse of the individual expected survival. This latter quantity and the general population other-cause mortality *λ*_*P*_ are derived from population life tables.

Among these non-parametric estimators, only the KM estimator is a purely cause-specific estimator because, in our case of cause-specific setting, it uses only cancer specific death information. Though used in the cause-specific setting, the wNA estimator uses also the population other-cause mortality to correct the estimation of net survival. The PP estimator is used only in population-based settings; it needs the other-cause mortality and the vital status to provide an estimate of net survival.

#### Parametric and semiparametric models

##### Cox model

In the cause-specific setting, the semiparametric Cox proportional hazards model expresses the excess hazard at time t as: *λ*_*E*_(*t*, *X*) = *λ*_*E*, 0_(*t*) exp(*β*^*T*^*X*) where *λ*_*E*, 0_(*t*), the baseline excess hazard at time *t*, and β corresponds to the proportional linear effect of covariate *X* on the baseline excess hazard estimated separately through the semiparametric approach.

One solution to derive the baseline cumulative excess hazard function from the Cox model was given by Breslow [20]. Using Breslow estimator with cancer death as status indicator *δ*_*i*_, the baseline cumulative excess hazard function Λ_*E*, 0_(*t*) may be estimated with the expression applied to times *t*_*i*_ at which the events take place:

$$ {\widehat{\Lambda}}_{\mathrm{E},0}\left(\mathrm{t}\right)=\sum \limits_{i=1}^n\frac{1\left({\mathrm{t}}_{\mathrm{i}}\le \mathrm{t}\right){\Delta}_{\mathrm{i}}}{\sum_{\mathrm{j}\in \mathrm{R}\left({\mathrm{t}}_{\mathrm{i}}\right)}{\mathrm{e}}^{\widehat{\upbeta^{\mathrm{T}}}{\mathrm{X}}_{\mathrm{j}}}} $$

In this equation, $\mathcal{R}(t)$ denotes the risk at time *t* of all individuals still at risk of death from cancer at time *t*, $\widehat{\upbeta}$ corresponds to the effect of covariates and *Δ*_*i*_ corresponds the indicator of death due to the cancer of interest. In this formula, we estimate unadjusted cumulative excess hazard by setting $\widehat{\beta}=0$ [21].

The corresponding net survival from Breslow’s estimator is therefore:

$$ {\widehat{S}}_{Cox_E}\left(t,X\right)=\sum \limits_i^n\exp \left(-{\widehat{\Lambda}}_{E,0}(t)\exp \left(\widehat{\beta^T}{X}_i\right)\right), $$

where ${\widehat{\varLambda}}_{E,0}$ corresponds to the baseline cumulative excess hazard function estimated separately from $\widehat{\beta}$.

##### The new flexible model

The new model is an extension of the flexible parametric excess hazard model proposed by Giorgi et al. [15]. It is based on the seminal excess hazard model of Estève et al. [14] where the observed hazard of a patient *i* at time *t*_*i*_ is:

$$ {\lambda}_O\left({t}_i|{X}_i\right)={\lambda}_{E,0}\left({t}_i\right)\exp \left[{\beta}^T{X}_i\right]+{\lambda}_{Pi}\left({t}_i|{Z}_i\right) $$

and where the baseline excess hazard (*λ*_*E*, 0_) is modelled by a piecewise constant function; *β* represents the effects of the vector of covariates *X* including demographic variables *Z* (such as age at diagnosis, year of diagnosis, sex, of the individual *i*).

In the model of Giorgi et al. [15], the baseline excess hazard and the time-dependent covariates are both modelled using specific B-spline functions. More precisely, for a higher degree of flexibility, Giorgi et al. used quadratic B-splines (order 3) and two interior knots.

For simplicity, only the case of proportional hazard effects of prognostic covariates is considered. The simplified version of Giorgi’s model is then:

$$ {\lambda}_O\left({t}_i|{X}_i\right)=\left[\sum \limits_{j=-2}^2{\nu}_j{\mathrm{B}}_{j,3}\left({t}_i\right)\right]\exp \left({\beta}^T{X}_i\right)+{\lambda}_P\left({t}_i|{Z}_i\right) $$

where *v*_*j*_ are the spline coefficients, *B*_*j*, 3_(*t*_*i*_) the value at time *t*_*i*_ of the *j* ^th^ B-spline of order 3 and degree 2, *X* the vector of covariates with proportional hazard effects *β*, and *λ*_*Pi*_ the other-cause hazard of individual *i*, at age *a*_*i*_ + *t*_*i*_ in year *y*_*i*_ + *t*_*i*_.

In agreement with Cheuvart and Ryan [19], we considered that the other-cause mortality of a participant in a clinical trial may be corrected by multiplying the population hazard obtained from the life table by a scale parameter *α*. This parameter is the average effect of selection on the other-cause mortality in the trial participants. This effect in the general population with same demographic characteristics equals 1 (*α* = 1). The new flexible model we call the “rescaled B-spline” model (RBS) can be written as follows:

$$ {\lambda}_O\left({t}_i|{X}_i\right)=\left[\sum \limits_{j=-2}^2{\nu}_j{\mathrm{B}}_{j,3}\left({t}_i\right)\right]\exp \left({\beta}^T{X}_i\right)+\alpha {\lambda}_P\left({t}_i|{Z}_i\right) $$

To estimate the parameters of the RBS model, we used the maximum likelihood procedure. The log-likelihood of the RBS model can be written:

$$ {l}_i\left(\beta, v,\alpha \right)=\sum \limits_{i=1}^n-\exp \left({\beta}_i{X}_i\right){\int}_0^t\left(\sum \limits_{j=-2}^2{\nu}_j{\mathrm{B}}_{j,3}\left({t}_i\right)\right) dt-{\alpha \Lambda}_p\left({t}_i|{Z}_i\right)+{\delta}_i\log \left({\lambda}_E\left({t}_i|{X}_i\right)+\alpha {\lambda}_P\left({t}_i|{Z}_i\right)\right) $$

where, given an individual *i*, observation *δ*_*i*_ = 1 is the indicator of death from any cause, *α* the scale parameter of the instantaneous other-cause mortality *λ*_*P*_, and Λ_*P*_ the cumulated value of *λ*_*P*_ over all the follow-up duration. The rescaled cumulative population hazard *α*Λ_*P*_ may not be a constant as in the classical additive excess hazard model. In addition, the scale parameter *α* will be considered in the estimation process. For mathematical convenience and because all patients may die from another cause than the cancer under study, it is assumed that *α* > 0 and constant over time. The maximization of the log-likelihood was performed using optim function in R based on Byrd method for non-linear optimization problems with box constraints [22]. The estimates of net survival were derived from the cumulative excess hazard calculated by derivation of the corresponding estimate of the excess hazard function. The confidence interval of the net survivals was obtained with a Monte-Carlo method [17]. The R code that implements these estimation procedures is available on request from the authors.

### The simulation study

We carried out a simulation study to assess the performance of the RBS model in estimating the net survival in clinical trials and compare this performance with those of previous models and estimators used in clinical trials.

#### The simulation design

The simulation considered a randomized clinical trial that would compare treatment vs. placebo effects. The French general-population life-table was used to construct a “corrected” life table that would correspond to other-cause mortality of trial participants. The “corrected” life table mortality rates were the mortality rates of the initial life table multiplied by the scale parameter *α*. This “corrected” life table was then used to generate *T*_*P*_, the individual time-to-death from another cause than cancer in a trial. For each patient, we generated also *T*_*E*_, the time-to-death from cancer and *T*_*C*_, the time to right-censoring (see the Data generation section). All the times *T*_*E*_, *T*_*P*_ and *T*_*C*_ were generated independently from each other. An individual observed time-to-death *T*_*O*_ was then the smallest of *T*_*P*_, *T*_*E*_, and *T*_*C*_. In addition, as in Grafféo et al. [23], from these generated times, we inferred a time-to-death *T*_*N*_ in the net survival setting where the patients would only die from cancer. Thus, *T*_*N*_ is the smallest of *T*_*E*_ and *T*_*C*_.

In this simulation, the causes of death were considered as known; thus, *T*_*N*_ and well-classified cancer-specific causes of death can be used with the KM estimator to obtain a gold standard (GS) estimator of the net survival; that is, the survival that would be obtained if cancer were the only possible cause of death (Table 1).

**Table 1:** Subset of simulated data in cause-specific and population-based settings

Patient	Observed data						Other simulated times-to-event				Data in the net survival setting	
	*T*_*O*_	Vital status	Death from cancer	0% misCoD	20% misCoD	30% misCoD	*T*_*P*_	*T*_*E*_	*T*_*C*_	Death from another cause	*T*_*N*_	Statu*s*_*N*_
1	4.61	1	0	0	0	1	4.61	11.27	15.00	1	11.27	1
2	9.21	1	1	1	1	1	63.78	9.21	15.00	0	9.21	1
3	7.83	1	1	1	1	0	65.06	7.83	15.00	0	7.83	1
4	12.47	1	1	1	0	1	58.17	12.47	15.00	0	12.47	1
5	15.00	0	0	0	0	0	52.64	25.12	15.00	0	15.00	0

We present a short excerpt to clarify the use of simulation data with each estimation method. Column “Patient” shows an identifier. The simulated data include: (i) observed data: time-to-death (***T***_***O***_), the vital status (1 if death from any cause, 0 alive), the cause of death (1 if due to cancer, 0 otherwise or alive). The table considers three degrees of cause-of-death misclassification (misCoD 30, 20, and 0% a highly improbable setting); (ii) simulated times-to-death: from other cause (*T*_*P*_), and from cancer (*T*_*E*_), the time from censoring (*T*_*C*_); (iii) data observed if cancer were the only possible cause of death: the time to death *T*_*N*_ (the smallest of *T*_*E*_ and *T*_*C*_) and the vital status in the net survival setting where cancer is the only possible cause of death

For example, the case of Patient 4, should be classified as death from cancer because the time-to-death from cancer (12.47 years) is lower than the time from censoring (15 years) and corresponds to the smallest of *T*_*P*_*, T*_*E*_ and *T*_*C*_. This case would have been classified as “death from other cause” (i.e., 0% misCoD = 0) if he were followed-up to 58.17 years. In Column “20% misCoD”, the cause of death is wrongly coded “death from other cause”. In the latter case, 20% of all cancer deaths are misclassified as deaths from other causes

Various scenarios were built by combining various values of *α* with various proportions of cause-of-death misclassification. Within each scenario, 1000 datasets of 1000 patients each were simulated.

#### Data generation

##### Adjusted excess mortality

In this simulation, for simplicity, the patients were considered to be of same sex. Trial group (placebo or treatment) covariates and age at diagnosis were independently generated. The treatment group was generated so as to obtain 50% of patients in each trial group. The age at diagnosis was obtained from a uniform distribution so as to obtain 25% of patients aged 24 to 45 years old, 50% aged 46 to 64 years, and 25% aged 65 to 70 years. We assumed a linear effect *β*_*age*_ = 0.05 for the effect of centered age on the excess hazard. The beneficial effect of the treatment on the excess hazard of death was *β*_*treatment*_ = − 0.5.

In all scenarios, a generalized Weibull distribution [23] was assumed for the distribution of the baseline excess hazards and the individual times-to-death from cancer *T*_*E*_ were generated using the inverse transformation method to account for covariate effects [23]. The time to right-censoring *T*_*C*_ was generated from a uniform distribution $\mathcal{U}\sim \left[0;b\right]$ with *b* chosen so as to obtain a censoring rate of 50%.

##### Rescaled other-cause mortality

*T*_*P*_, the time-to-death from another cause (i.e., the other-cause mortality in the general population multiplied by a scale parameter that depends on the characteristics of the trial participants) was generated using the French life table survexp.fr available in the eponymous R package [24]. This life table is stratified by age, sex, and year of cancer diagnosis. For each patient *i*, the “rescaled” other-cause mortality rate was considered as *αλ*_*P*_(*a*_*i*_ + *t*_*i*_, *Z*_*i*_), where *α* is the selection effect and *λ*_*P*_(*a*_*i*_ + *t*_*i*_) the general population other-cause mortality at age *a*_*i*_ + *t*_*i*_ adjusted on demographic covariates *Z* as derived from the life table.

Four scenarios were considered for the other-cause mortality of cancer patients in clinical trials: (1) patients comparable to the general population in terms of other-cause mortality (*α* = 1); (2) patients more robust than the general population (*α* = 0.5); (3) patients frailer than the general population (*α* = 2); and (4) patients much more frail than the general population (*α* = 4).

##### Misclassification of the cause of death

Three conditions were considered regarding the proportion of errors in identifying the causes of death. These conditions are useful to compare cause-specific with population-based methods in realistic settings. Indeed, in many clinical trials with medium- to long-term follow-ups (10 to 15 years), it is difficult to obtain accurate causes of death. The simulation considered three cause-of-death misclassification rates: (i) 0%, a rare condition where all information on cancer-related death is true; (ii) 20% of misclassification beyond 5 years of follow-up, which means that 20% of deaths from cancer are wrongly attributed to another cause; and (iii) 30% of misclassification beyond 5 years of follow-up. Actually, in active follow-ups in clinical trials, the causes of death over short-term follow-ups (e.g., 5 years) are rather reliable.

To sum up, Scenarios 1 to 4 and conditions i to iii were designed to: a) assess the performance of the estimators in case of no misclassification and no selection effect; b) assess the bias due to misclassification with cause-specific approaches and examine then the interest of population-based estimators; c) assess the performance of the new RBS model in correcting for selection bias alone; and, d) assess the performance of the cause-specific methods in presence of selection effect and misclassification.

#### Performance criteria

The theoretical net survival in each of the treatment and the placebo group is the average of the individual net survivals. Thus, the theoretical net survival can be written:

$$ {S}_{\mathrm{E},\mathrm{j}}(t)=\frac{1}{n_j}\sum \limits_{i=1}^{n_j}\exp \left[-{\Lambda}_0(t)\exp\ \left({\beta}_{age}\ast {age}_{ij}+{\beta}_{treatment}{Z}_{ij}\right)\right] $$

*n*_*j*_ is the number of patients in each group *j* and Λ_0_(*t*) is the excess cumulative baseline hazard from the generalized Weibull distribution.

The performance in estimating the net survival is established on: (1) the bias $\frac{1}{m}\sum \limits_{j=1}^m{\widehat{S}}_{M,E,j}(t)-{S}_{\mathrm{E},\mathrm{j}}(t)$, where ${\widehat{S}}_{M,E,j}(t)$ is the mean of net survival estimates by model or estimator *M* at time *t* in group *j*, *S*_E, j_(*t*) is the theoretical net survival at time *t* in group *j*, and *m* is the number of simulations; (2) the relative bias (R. Bias) $\left(\frac{\frac{1}{m}\sum \limits_{j=1}^m\ {\widehat{S}}_{M,E,j}(t)-{S}_{\mathrm{E},\mathrm{j}}(t)}{S_{\mathrm{E},\mathrm{j}}(t)}\right)\ast 100$; (3) the root mean square error $\sqrt{\frac{1}{m}\sum \limits_{j=1}^m{\left({\widehat{S}}_{M,E,j}(t)-{S}_{\mathrm{E},\mathrm{j}}(t)\ \right)}^2}$; (4) the empirical coverage rate (ECR); i.e., the proportion of samples in which the 95% confidence interval of the estimated net survival at time *t* in group *j* contains *S*_E, j_(*t*). These statistical indicators were calculated at 5, 10, and 15 years of follow-up. We also calculated the performances of covariates (centered age and treatment) effects estimated by Cox and RBS models using bias, root mean square error and ECR.

## Results

### Simulation results

**Fig. 1:**
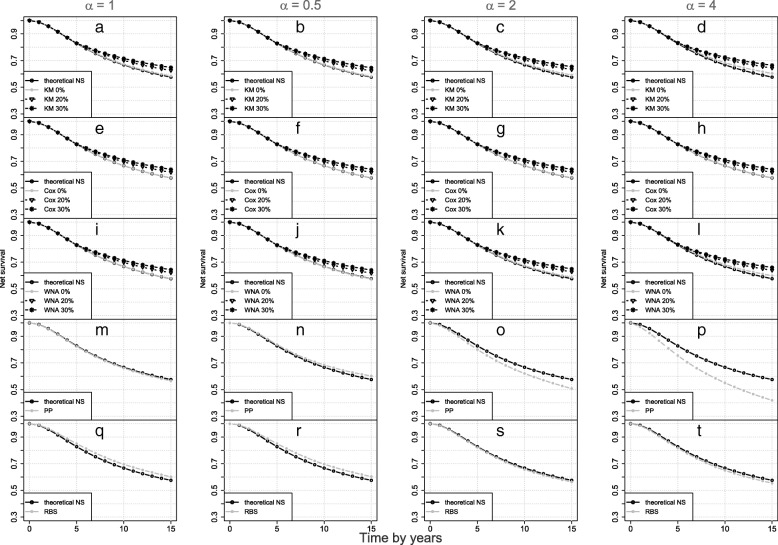
Gold standard (GS) net survival distribution and those estimated by the KM, the Cox and the wNA methods without and with misclassification of the cause of death (0, 20 and 30%) with selection effect equals 1, 0.5, 2 and 4. Each panel compares one method to the GS: (**a**)-(**d**) for KM; (**e**)-(**h**) for Cox; (**i**)-(**l**) for WNA; (**m**)-(**p**) for PP; (**q**)-(**t**) for RBS

In this section, we present the simulation results. We provide only the results relative to the placebo group (because the effects of cause-of-death misclassification were not different between the treatment and the placebo group) and at 5, 10, and 15 years of follow-up. The performance criteria of each method by scenario are shown in Table 2. The corresponding net survival curves are presented in Fig. 1. The results relative to the estimation of Cox and RBS models’ parameters are shown in Table 3. The boxplots of Cox and RBS models’ parameters estimated in all scenarios are presented in Additional files 1, 2 and 3.

**Table 2:** Performance in terms of net survival of various methods in various scenarios

Method & Misc.	Bias × 100	5-year net survival			Bias ×100	10-year net survival			Bias ×100	15-year net survival		
		R.Bias	RMSE	ECR		R.Bias	RMSE	ECR		R.Bias	RMSE	ECR
*Scenario 1 (α = 1)*												
Gold standard	0.012 (*0.827* ^a^)	0.0145	0.012	94.7	0.017 (*0.667* ^a^)	0.254	0.015	95.0	0.025 (*0.575* ^a^)	0.043	0.016	95.2
CSS methods												
KM & 0%	0.126	0.152	0.012	94.5	0.434	0.650	0.016	93.5	0.778	1.353	0.018	92.8
KM & 20%	0.126	0.152	0.012	94.5	3.296	4.941	0.036	42.9	4.993	8.683	0.052	13.8
KM & 30%	0.126	0.152	0.012	94.5	4.803	7.200	0.050	10.2	7.245	12.60	0.074	0.4
Cox & 0%	0.036	0.043	0.011	95.7	0.036	0.053	0.015	97.6	0.046	0.080	0.016	97.7
Cox & 20%	0.036	0.043	0.011	95.7	2.869	4.301	0.032	60.6	4.144	7.206	0.041	38.5
Cox & 30%	0.036	0.043	0.011	95.7	4.364	6.54	0.045	23.8	6.366	11.071	0.065	4.7
wNA & 0%	0.027	0.032	0.012	94.4	0.089	0.133	0.015	94.6	0.143	0.248	0.016	94.3
wNA & 20%	0.027	0.032	0.012	94.4	2.991	4.484	0.033	50.7	4.432	7.707	0.047	21.3
wNA & 30%	0.027	0.032	0.012	94.4	4.520	6.776	0.047	14.8	6.726	11.697	0.069	1.8
PNS methods												
PP	−0.359	−0.434	0.014	94.5	−0.574	−0.860	0.017	95.9	−0.999	−1.737	0.020	94.0
RBS	−0.102	−0.123	0.015	94.2	−0.445	−0.667	0.025	92.3	−0.459	−0.790	0.037	93.1
*Scenario 2 (α = 0.5)*												
Gold standard	−0.005 (*0.787* ^a^)	− 0.006	0.011	95.4	0.022 (*0.644* ^a^)	0.034	0.013	96.8	0.048 (*0.576* ^a^)	0.083	0.014	96.7
CSS methods												
KM & 0%	0.061	0.077	0.011	95.3	0.234	0 .363	0.014	96.7	0.437	0.758	0.015	94.7
KM & 20%	0.061	0.077	0.011	95.3	3.111	4.830	0.033	42.9	4.702	8.163	0.049	13.8
KM & 30%	0.061	0.077	0.011	95.3	4.636	7.198	0.048	9.5	6.984	12.449	0.071	0.4
Cox & 0%	0.033	0.041	0.010	97.2	0.041	0.063	0.013	97	0.071	0.123	0.014	98.0
Cox & 20%	0.033	0.041	0.010	97.2	2.876	4.465	0.031	65.6	4.173	7.244	0.044	44.6
Cox & 30%	0.033	0.041	0.010	97.2	4.377	6.796	0.045	30.4	6.404	11.118	0.065	7.0
wNA & 0%	−0.038	−0.048	0.011	95.3	−0.113	−0.175	0.014	96.8	−0.210	−0.364	0.014	97.2
wNA & 20%	−0.038	−0.048	0.011	95.3	2.804	4.354	0.033	50.5	4.130	7.170	0.047	23.8
wNA & 30%	−0.038	−0.048	0.011	95.3	4.351	6.756	0.045	14.8	6.458	11.21	0.066	1.8
PNS methods												
PP	0.897	1.139	0.015	88.0	1.731	2.687	0.022	82.9	2.618	4.545	0.031	67.4
RBS	−0.023	−0.029	0.012	94.9	−0.303	− 0.470	0.019	91.8	−0.251	− 0.435	0.028	90.1
*Scenario 3 (α = 2)*												
Gold standard	0.008 (*0.827* ^a^)	0.009	0.012	95.6	0.016 (*0.667* ^a^)	0.023	0.015	95.9	0.057 (*0.575* ^a^)	0.099	0.016	95.7
CSS methods												
KM & 0%	0.233	0.281	0.012	94.3	0.818	1.226	0.017	93.2	1.513	2.631	0.023	87.2
KM & 20%	0.233	0.281	0.012	94.3	3.624	5.433	0.039	38.7	5.611	9.758	0.058	11.7
KM & 30%	0.233	0.281	0.012	94.3	5.116	7.670	0.053	8.8	7.830	13.617	0.079	0.2
Cox & 0%	0.036	0.043	0.011	97.3	0.062	0.092	0.015	98.5	0.113	0.196	0.017	98.5
Cox & 20%	0.036	0.043	0.011	97.3	2.877	4.313	0.032	65.1	4.175	7.260	0.044	45.1
Cox & 30%	0.036	0.043	0.011	97.3	4.347	6.517	0.045	29.4	6.386	11.106	0.065	7.3
wNA & 0%	0.136	0.164	0.012	94.5	0.484	0.725	0.016	95.0	0.919	1.598	0.020	94.5
wNA & 20%	0.136	0.164	0.012	94.5	3.329	4.991	0.036	44.3	5.087	8.846	0.053	18.1
wNA & 30%	0.136	0.164	0.012	94.5	4.842	7.259	0.050	13.0	7.347	12.777	0.075	0.8
PNS methods												
PP	−2.784	−3.366	0.031	50.7	−4.693	−7.035	0.049	27.1	−6.831	−11.88	0.070	8.2
RBS	−0.284	−0.343	0.021	93.4	−0.667	−1.000	0.036	92.7	−0.668	−1.161	0.053	94.0
*Scenario 4 (α = 4)*												
Gold standard	0.012 (*0.727* ^a^)	0.015	0.012	95.1	0.082 (*0.644* ^a^)	0.127	0.016	95.8	0.083 (*0.575* ^a^)	0.144	0.017	95.8
CSS methods												
KM & 0%	0.493	0.626	0.013	93.2	1.633	2.535	0.023	80.6	2.812	4.890	0.034	66.0
KM & 20%	0.493	0.626	0.013	93.2	4.335	6.731	0.046	25.2	6.775	11.782	0.070	6.1
KM & 30%	0.493	0.626	0.013	93.2	5.754	8.934	0.059	6.7	8.867	15.42	0.090	0.3
Cox & 0%	0.037	0.047	0.011	97.3	0.077	0.105	0.016	97.5	0.047	0.081	0.019	97.7
Cox & 20%	0.037	0.047	0.011	97.3	2.894	4.493	0.033	67.5	4.127	1.177	0.045	53.0
Cox & 30%	0.037	0.047	0.011	97.3	4.369	6.784	0.046	31.2	6.329	11.006	0.065	12.6
wNA & 0%	0.397	0.504	0.013	93.7	1.315	2.041	0.021	85.0	2.276	3.958	0.030	74.3
wNA & 20%	0.397	0.504	0.013	93.7	4.054	6.295	0.043	30.7	6.304	10.963	0.065	9.1
wNA & 30%	0.397	0.504	0.013	93.7	5.492	8.527	0.057	8.6	8.428	14.657	0.086	0.6
PNS methods												
PP	−7.274	−9.268	0.074	0.1	−11.690	18.152	0.118	0.00	−15.629	−27.173	0.157	0.00
RBS	−0.469	−0.595	0.0311	93.1	−0.869	−1.349	0.0532	93.6	−0.934	−1.624	0.0762	93.8

a The true net survival values are provided at 5, 10, and 15 years of follow-up for each scenarios

*Misc* misclassification rate , *RMSE* root mean square error, *ECR* empirical coverage rate, *CSS* cause-specific survival, *KM* Kaplan-Meier, *wNA* weighted Nelson-Aalen, *PNS* population-based net survival, *PP* Pohar-Perme, *RBS* rescaled B-spline, *R.Bias* relative bias

**Table 3:** Performance in terms of parameters estimation (${\widehat{\beta}}_{age},{\widehat{\beta}}_{treatment},\kern0.5em \widehat{\boldsymbol{\alpha}}\Big)$ with rescaled B-spline (RBS) and Cox model in various scenarios

Method & Misc.	*Scenario 1 (α = 1)*			*Scenario 2 (α = 0.5)*			*Scenario 3 (α = 2)*			*Scenario 4 (α = 4)*		
	RBias	RMSE	ECR	RBias	RMSE	ECR	RBias	RMSE	ECR	RBias	RMSE	ECR
${\widehat{\beta}}_{age}$*(β*_*age*_ = 0.05*)*												
CSS methods												
Cox & 0%	0.528	0.392	94.5	0.360	0.388	95.7	0.170	0.389	94.8	0.802	0.388	94.8
Cox & 20%	0.552	0.392	94.4	0.355	0.389	96.3	0.128	0.391	95.2	0.709	0.389	94.6
Cox & 30%	0.612	0.392	94.7	0.446	0.388	96.1	0.141	0.391	95.1	0.832	0.389	94.6
PNS methods												
RBS	−0.489	0.444	96.4	−1.749	0.295	97.1	0.417	0.686	94.04	2.006	1.065	91.7
${\widehat{\beta}}_{treatment}$(*β*_*treatment*_ = − 0.5)												
CSS methods												
Cox & 0%	0.869	0.392	94.8	−0.075	0.388	95.5	0.245	0.389	94.8	−0.311	0.388	93.9
Cox & 20%	1.027	0.392	95.2	0.042	0.389	95.3	0.710	0.391	94.6	−0.044	0.389	93.7
Cox & 30%	0.820	0.392	94.7	−0.271	0.388	95.3	0.773	0.391	95.8	0.095	0.389	94.3
PNS methods												
RBS	1.920	0.234	95.6	3.209	0.155	97.1	0.774	0.391	95.8	−1.083	1.032	92.3
$\widehat{\alpha}$	0.060	0.444	92.6	15.754	0.305	90.8	−5.863	0.696	93.6	−7.419	1.105	94.9

*Misc* Misclassification rate, *RMSE* root mean square error, *ECR* empirical coverage rate, *CSS* cause-specific survival, *PNS* population-based net survival, *RBS* rescaled B-spline, *RBias* relative bias

#### No selection effect and no cause-of-death misclassification (scenario 1a)

Scenario 1a allowed evaluating the performance of cause-specific vs. population-based methods in a theoretical setting. As expected, the estimates of net survival obtained with all methods had very small bias. Whatever the estimator, the ECR was close to 95%. In comparison with the root mean square error (RMSE) obtained with the GS (KM estimator applied to data where patients would only die from cancer, i.e. net survival setting), the RMSEs of the KM, Cox, and wNA estimators were similar, whereas the RMSEs obtained with population-based methods were slightly higher. For example, at 15 years follow-up, the RMSEs were: 0.016 with GS, 0.018 with KM, 0.016 with Cox, 0.016 with wNA, 0.020 with PP, and 0.036 with RBS). Otherwise, Cox and RBS parameters estimates had good performances and globally better in Cox than RBS model (Table 3).

#### No selection effect but presence of cause-of-death misclassification (scenario 1b)

As expected, increasing the proportion of cause-of-death misclassification resulted in increased biases with KM and wNA estimators. With 30% misclassification, the differences in terms of bias between the GS and each of KM, Cox, and wNA were, respectively, 0.072, 0.063 and 0.067. The ECRs with KM, Cox, and wNA estimators were close to 0 when the misclassification was 30%. With 30% misclassification, the differences in RMSE between GS and each of KM, Cox, and wNA estimates at 15 years follow-up were, respectively, 0.058, 0.049, and 0.053. As the population-based methods did not use the cause of death information, we can compare directly their results with those of cause-specific methods. In this scenario, the performances of the PP and the RBS estimator were better than those obtained with the cause-specific methods in case of no selection effect (α = 1) and 20 to 30% misclassification.

In summary, in Scenario 1 and with *α*= 1, the performance of the RBS regression model in net survival estimation was better than that of another model. However, Cox model parameters (effect of age and treatment) estimates were very slightly impacted in terms of relative bias, RMSE and ECR (Table 3).

#### Presence of selection effect but no cause-of-death misclassification (scenarios 2a, 3a, 4a)

With cause-specific methods KM and wNA, the bias in the estimate of the net survival increased with the increase of α (the effect of selection). The bias with the RBS was much more important than with the GS. The bias with PP was much more important than with the GS. The estimates of net survival obtained with the RBS model had higher RMSEs than those obtained with wNA or KM and the estimates obtained with PP had higher values than those obtained with the other methods. The ECRs obtained with KM and wNA were far from 95%, the nominal value; they approached zero with PP with the increase of the selection effect; i.e., with the increase of α values over 1. The ECRs obtained with Cox and RBS models remained stable and close to their nominal values (Table 2). Besides, Cox model parameters (effect of age and treatment) estimates were not impacted in terms of relative bias, RMSE, ECRs than that of RBS (Table 3).

#### Presence of selection effect and cause-of-death misclassification (scenarios 2b, 3b, 4b)

In Scenarios 2 to 4 with 20% or 30% cause-of-death misclassification, the biases in the estimates of net survival obtained with each of KM, Cox, and wNA were 3 to 4 times more important than those obtained with the GS. The biases obtained with the RBS model did not change much. The ECRs of the estimate of net survival obtained with KM, Cox, or wNA was close to 0 when the cause-of-death misclassification was 30%. With 30% misclassification at 15 years, the differences between the RSME obtained with GS and each of the tree cause-specific methods (KM, Cox, wNA) were 0.073, 0.048 and 0.069 (Scenario 4b). However, Cox model parameters (effect of age and treatment) estimates were slightly impacted in terms of relative bias, RMSE, ECRs than that of RBS.

### Application to clinical trial data

To illustrate the interest of the RBS model in estimating net survival in clinical trials and be able to compare it with other approaches, we used data from a clinical trial that included 506 prostate cancer patients [25] (USA, 1967–1969, data published by Andrews and Herzberg [26]). The patients were randomly allocated to one of four treatment regimens: placebo or 0 mg/d, 0.2 mg/d, 1 mg/d, and 5 mg/d of per os diethylstilbestrol. As in previous works on these data, we gave indicator 0 to the low-doses (0 and 0.2 mg/d) and 1 to high-doses (1 and 5 mg/d) and considered seven other covariates: age, weight index, performance rating, history of cardiovascular disease, serum hemoglobin, size of primary lesion, and Gleason stage/grade category [25]. However, in the parametric model as in the simulation study, age was centered on the median age (73 years). Because 23 patients have missing information, 483 patients were kept for analysis: 241 in the low-dose and 242 in the high-dose treatment group. Concerning the stage variable, 278 patients were in stage 3 and 205 in stage 4. The median follow-up was about 65 months and the whole follow-up was 76 months.

In estimating the net survival, population-based methods used the USA life tables from 1967 to 1973 that included covariates age, sex, and year of diagnosis. The distributions of the two treatment groups were compared using a specific log-rank-type test for net survival comparisons [23] and considering *p* < 0.05 as significance level.

**Fig. 2:**
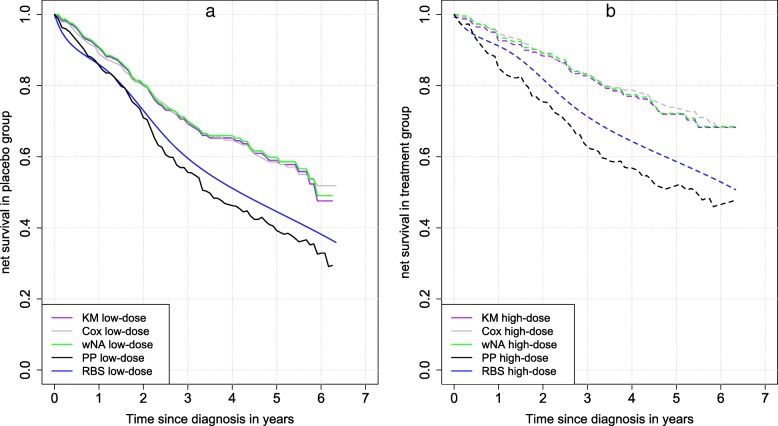
Distributions of net survival estimations carried out with cause-specific versus population-based methods (KM, Cox, and wNA vs. PP and RBS) in prostate cancer patients who received: (**a**) low-dose (solid line) diethylstilbestrol (0 and 0.2 mg/d); (**b**) high-dose (dashed line) diethylstilbestrol (1 and 5 mg/d)

The parametric cause-specific Cox and the proposed RBS models were used to estimate the effect of treatment on the excess hazard of death from prostate cancer. The net survival estimates over time by KM, wNA, PP and RBS methods in placebo group and in treatment group for all the patients are shown respectively in Fig. 2.a and Fig. 2.b. The log-rank-type test did not find a statistically significant difference in net survival estimates by PP method between the low-dose and the high dose group (${\chi}_1^2=3.61,\kern0.5em \mathrm{p}=0.057$). At the end of the 76-month follow-up period, the estimates of net survival in the high-dose group were: *S*_*KM*_ = 0.682, *S*_*Cox*_ = 0.662,  *S*_*wNA*_ = 0.684, *S*_*PP*_ = 0.478, and *S*_RBS_ = 0.565. The estimates in the low-dose group were: *S*_*KM*_ = 0.475, *S*_*Cox*_ = 0.498,  *S*_*wNA*_ = 0.491,  *S*_*PP*_ = 0.293, and *S*_RBS_ = 0.396. Table 4. shows results obtained with the two parametric Cox and RBS model adjusted for the covariates. Both models concluded to a significant reductive effect of high-dose treatment on the excess hazard of death from prostate cancer and to a higher estimated effect with the RBS model vs. Cox model (Excess hazard ratio, ${EHR}_{RB{S}_{high}- dose}=0.62\ \left[0.40;0.96\right]$) vs. ${EHR}_{CO{X}_{high}- dose}=0.43\ \left[0.30;0.64\right]$) . The RBS model showed that other-cause mortality in the trial participants was 1.51 times that of the US general population. Thus, on average, the expected mortality provided by the US life table is 1.51 times lower compared to the appropriate other-cause mortality in the trial participants. Correcting for both possible cause-of-death misclassification and effect of selection indicated that treatment effect (excess risk reduction = 38%) was lower than using a cause-specific Cox (excess risk reduction = 57%).

**Table 4:** Excess hazard ratios with Cox and RBS model. Results of the application on prostate cancer data

Variable & modalities	Cox model	RBS model
	EHR [95% CI]	EHR [95% CI]
*Treatment*		
High-dose DES	0.43 [0.30; 0.64]	0.62 [0.40; 0.96]
Low-dose DES		
*Age (centered)*	0.97 [0.95; 1.00]	0.98 [0.95; 1.01]
*Weight index* ^*a*^		
< 80	1.45 [0.75; 2.81]	2.05 [1.04; 4.05]
80–99	1.29 [0.87; 1.92]	1.73 [1.13; 2.66]
≥100		
*Performance rating*		
Limited activity	1.49 [0.87; 2.52]	1.62 [0.98; 2.66]
Normal activity		
*History of cardiovascular disease*		
Yes	0.86 [0.58; 1.28]	1.70 [1.15; 2.52]
No		
*Haemoglobin*		
< 9 g/100 mL	9.13 [3.64; 22.88]	5.66 [2.12; 15.09]
9–12 g/100 mL	1.01 [0.64; 1.59]	1.32 [0.86; 2.01]
≥12 g/100 mL		
*Size of primary lesion*		
≥30 cm^2^	4.02 [2.62; 6.16]	3.25 [1.91; 5.50]
< 30 cm^2^		
*Gleason stage/grade category*		
> 10	9.58 [5.50; 16.67]	2.22 [1.31; 3.77]
≥10		
*Selection effect (* $\widehat{\alpha}$ *)*		1.51 [0.89; 2.56]

^a^Weight (kg) - Height (cm) + 200 - *RBS* Rescaled B-spline model, *CI* confidence interval, *EHR* excess hazard ratio, *DES* diethylstilbestrol – α: parameter of the RBS model used to rescale the all-cause mortality in participants in a clinical trial

## Discussion

The present work proposes a new flexible population-based model to estimate long-term net survival in clinical trials. To account for biases due to cause-of-death misclassification and patient selection, the model extends the excess hazard models developed by Estève et al. [14, 15] or Giorgi et al. [14, 15] and considers settings where the other-cause mortality is different from that of the general population of same general characteristics.

One advantage of the new RBS model is that it corrects the other-cause mortality of trial participants and provides more precise estimates of the excess hazard and the net survival in presence of selection. Simulation study has shown that the RBS model provides accurate estimates of the net survival. In the application, the RBS model showed that, due to selection, the other-cause mortality of trial participants was, on average, 1.51 times that of the general population, and the treatment effect was lower, compare to estimation obtained using cause-specific approach. This selection problem was already mentioned by Augustin et al. [27] who found that the effects of recent therapeutics on net survival in mantle-cell lymphoma patients included in a clinical trial were different from those seen in a larger group of patients found in cancer registries and concluded that trial patients are highly selected and may not be representative of the patients encountered in everyday practice. However, in accordance with previous comments on Augustin’s study [28], the present simulation study shows that some standard population-based methods, such as the PP estimator used by Augustin, provide biased estimates of net survival in the presence of a selection effect. Within this context, RBS model may allow for selection and provide more accurate comparisons between trial and other patients’ survivals.

Another advantage of the RBS model is that it is more flexible than that of Cheuvart and Ryan [19] in estimating the baseline excess hazard function. Estimating cancer excess hazard with the RBS model by a flexible function instead of a step function (that represents only discontinuous constant excess mortality rates) is more realistic and reliable in an epidemiological or clinical setting. Contrarily to cause-specific methods, the RBS, as other population-based methods, does not require knowing the cause of death and is thus insensitive to the cause-of-death misclassification. In the application, the cause of death information was used with cause specific methods (KM, Cox, and wNA) without any indication about its accuracy and the vital status was used with the PP estimator and the RBS model. Also, our results from the application have shown that net survival estimates with cause-specific methods (KM, Cox and wNA) were higher compared to that PP and RBS. Due to the robustness of the RBS model to estimate net survival in presence of selection effect, we are more confident with the RBS model estimates of net survival although the follow-up in the trial was less than 10 years. Indeed, net survival is probably overestimated using cause-specific methods because of an underestimation of the prostate cancer-related death. Besides, the misclassification phenomenon is not rare in prostate cancer data because of treatment impact [29]. The RBS model allowed rescaling the estimate of the net survival in treatment and placebo groups.

Cause-specific methods performed better than population-based methods in the absence of cause-of-death misclassification and/or selection effect. However, 0% misclassification is highly improbable; even in short-term clinical trials, there is always some proportion of cause-of-death misclassification because of information unreliability [7] or because of competing causes of death [5].

KM, wNA, and Cox model showed similar performances in estimating net survival in the absence of cause-of-death misclassification. In fact, KM and Cox model should be avoided because the assumption of independence between the other-cause and cancer mortality is not met; actually, some variables, such as age, may affect both mortalities. Thus, censoring death from other cause than cancer could be informative. Likewise, Pohar-Perme et al. [12] have shown that only the Nelson-Aalen estimator is consistent (asymptotically unbiased) in estimating the net survival using the inverse probability of censoring weighting. Therefore, the selection bias impacts obviously the cause-specific survival estimates with wNA because incorrect life tables are used to calculate the weights. In a trial, when the other-cause mortality of the participants is higher than that of the general population, the net survival estimate that uses a general population life table is underestimated, and inversely [28]. These results are in agreement with those of Baili et al. [30] and Stroup et al. [31]. For prostate cancer, Stroup et al. showed that prostate cancer patients with early stage have better health status than US general population. In addition, they also found that cause-specific methods are preferable to estimate net survival compared to population-based survival for prostate cancer patients with early diagnosis. These same conclusions were also found by Skyrud et al. [8] in Norway cancer registry among prostate cancer patients. However, in our application, the proposed model found that the selected patients were more frail than US general population. This can be explained by the fact that patients included in this trial had an advanced prostate tumor (stage 3 and 4) and were also more exposed to comorbidities due to their high median age.

In this work, we considered identical degrees of cause-of-death misclassification in the treatment and the placebo group, which is a plausible assumption in real clinical trials with long follow-up durations. This may explain the similar performances of cause-specific methods in the treatment and the placebo group in case of 20 or 30% cause-of-death misclassification after a five-year follow-up. However, as in population-based studies, and despite long-term follow-ups, distinct degrees of misclassification between the treatment and the placebo group are also possible. Some authors have shown that identical degrees of misclassification had less impact than distinct misclassifications on net survival estimation [9, 12].

Furthermore, Morisot et al [32] investigated the interest of multiple imputation approach in the estimation of cause-specific survival notably when a subset of cause of death was available. In their case there is a confidence in cause of death classification of some patients contrary to our case where we assume it exists an overall uncertainty on cause of death classification. Up to 50% missing values in the “cause of death” variable, Morisot et al have recommended multiple imputation method to obtain accurate estimates of cause-specific survival, notably in not large database. Indeed, this approach may be time-consuming and not satisfactory if a representative percentage of causes of death is not validated by experts. However, it is well-know that cause of death information may be difficult to be validate in long-term follow-up without autopsy. The consequence could be finally a high risk for misclassification of cause of death up to 68.2% [33], resulting in bias on net survival estimates using cause-specific method even after multiple imputation approach.

Despite its advantages, the RBS model has the limitations of most parametric excess-hazard models because of the assumptions regarding the baseline excess hazard and the effects of the covariates. For example, the effect of selection on the general population mortality was assumed to be multiplicative; this assumption is reasonable from an epidemiological or clinical point of view but may not be always met [19]. Another clinically plausible assumption would be to consider a non-proportional effect. Also, one may consider a heterogeneous selection effect between trial centers or individuals. For example, in the application, the effect of selection may be considered different between hospitals of the Veteran’s Administration, and the use of a frailty model for the risk of other-cause death could improve the RBS model. Within this context, the works of Zahl that account for heterogeneity in the competing risk model may improve the RBS model [34].

Besides, one assumption for net survival estimation is that *T*_*P*_ and *T*_*E*_ are conditionally independent on a set of explanatory covariates [14]. This assumption may not be verified and that raised some issues as well as in the classical framework of competing risks [35] and in the net survival setting resulting in informative censoring bias [17, 36]. In latter setting we have also to consider that general population life tables exist for each combination of demographic covariables, and observed data contain these demographic covariables. Indeed, the demographic covariables acts on both the excess hazard and the population hazard. However, these covariables are not always available on data or on general population life tables. The impact of their absence has been showed in Grafféo et al. [37], Danieli et al. [17] and studied by Pavlik and Pohar-Perme [38]. As shown by Danieli et al. [17] a regression model for excess hazard modelling adjusted on demographic covariables when there are present can deal with the informative censoring problem. In the absence of some important covariables the proposed model can be used to rescale the population hazard and offers potential research opportunities.

## Conclusion

In conclusion, the new RBS model allows estimating net survival in clinical trials. It corrects the biases of cause-of-death misclassification and of selection effect on the expected mortality in the general population. This makes it particularly useful in clinical trials with long follow-ups. With the RBS model, the researcher obtains accurate estimates of the excess hazard and, therefore, of net survival; however, he/she should check the strong assumption of homogeneous selection. Finally, the RBS model paves the way for new methodological developments in the field of net survival methods in multicenter clinical trials.

## Additional files

Additional file 1: Boxplots of the effect of centered age estimated with the RBS and Cox models’ in the simulation study. (PDF 27 kb)

Additional file 2: Boxplots of the effect of treatment estimated with the RBS and Cox models’ in the simulation study. (PDF 28 kb)

Additional file 3: Boxplots of the selected effect estimated with the RBS model in the simulation study. (PDF 17 kb)

## Abbreviations

ECR: Empirical coverage rate
EHR: Excess hazard rate
GS: Net survival setting or Gold Standard net survival
KM: Kaplan-Meier estimator
PP: Pohar-Perme estimator
RBS: Rescaled B-spline model
RMSE: Root Mean Square Error
wNA: Weighted Nelson-Aalen estimator
