# Supplementary figures and images for: Correcting for misclassification and selection effects in estimating net survival in clinical trials
Source: BMC Med Res Methodol. 2019 May 16;19:104. doi: 10.1186/s12874-019-0747-3 (PMC6524224; doi:10.1186/s12874-019-0747-3)

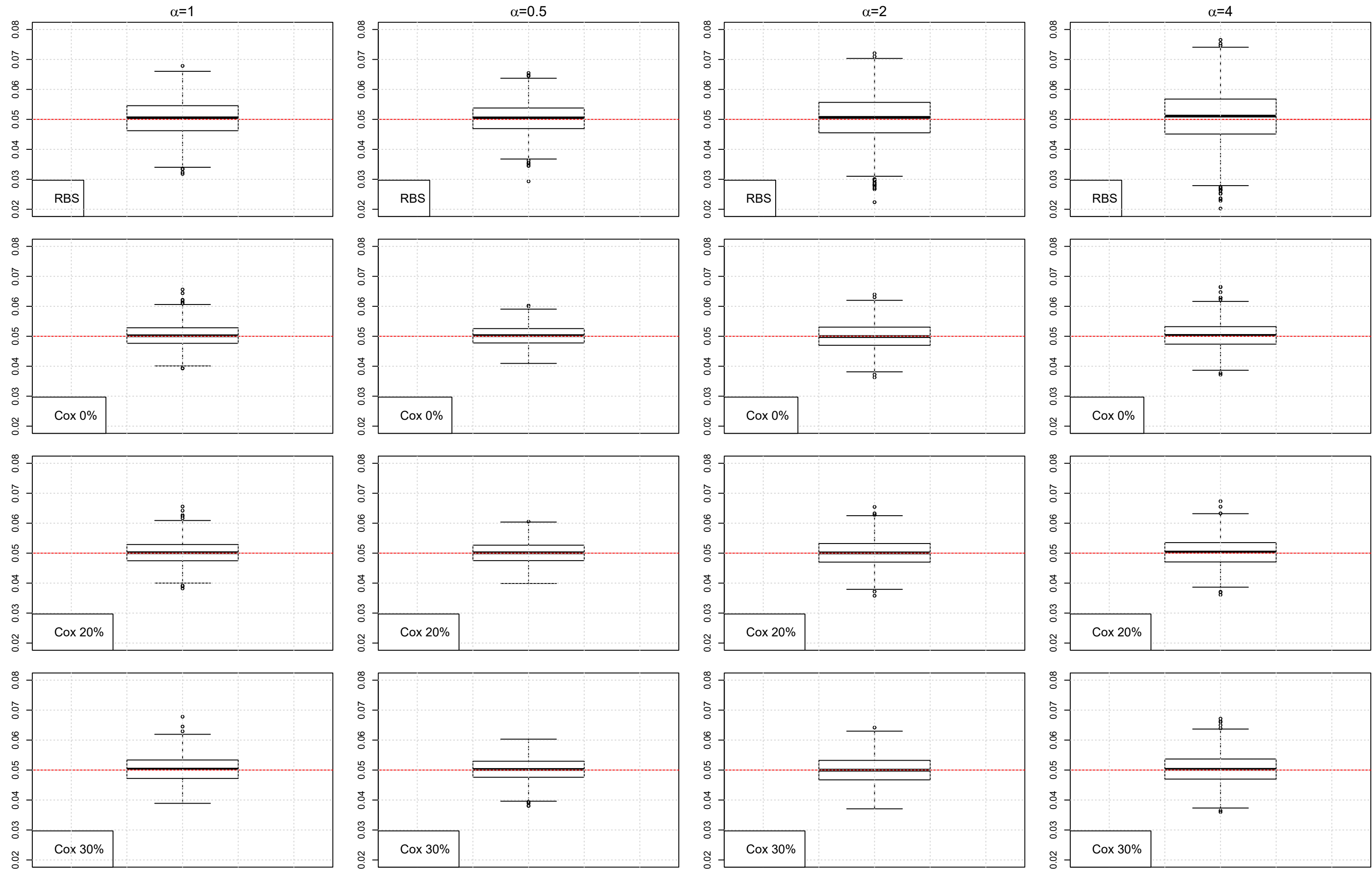

Supplement: Supplementary file 1 — Boxplots of the effect of centered age estimated with the RBS and Cox models’ in the simulation study. (PDF 27 kb) [file 12874_2019_747_MOESM1_ESM.pdf]

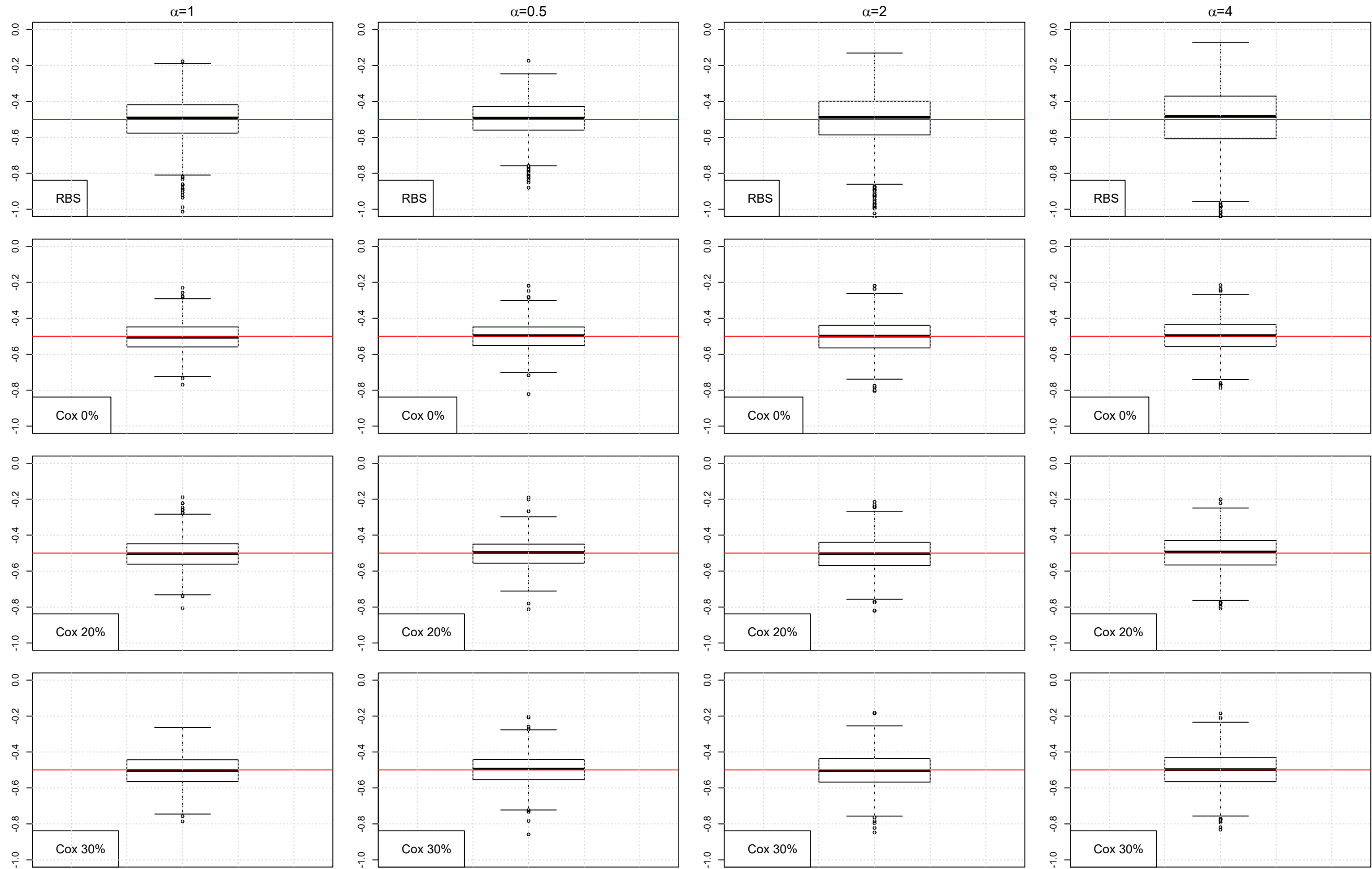

Supplement: Supplementary file 2 — Boxplots of the effect of treatment estimated with the RBS and Cox models’ in the simulation study. (PDF 28 kb) [file 12874_2019_747_MOESM2_ESM.pdf]

$\alpha=1$

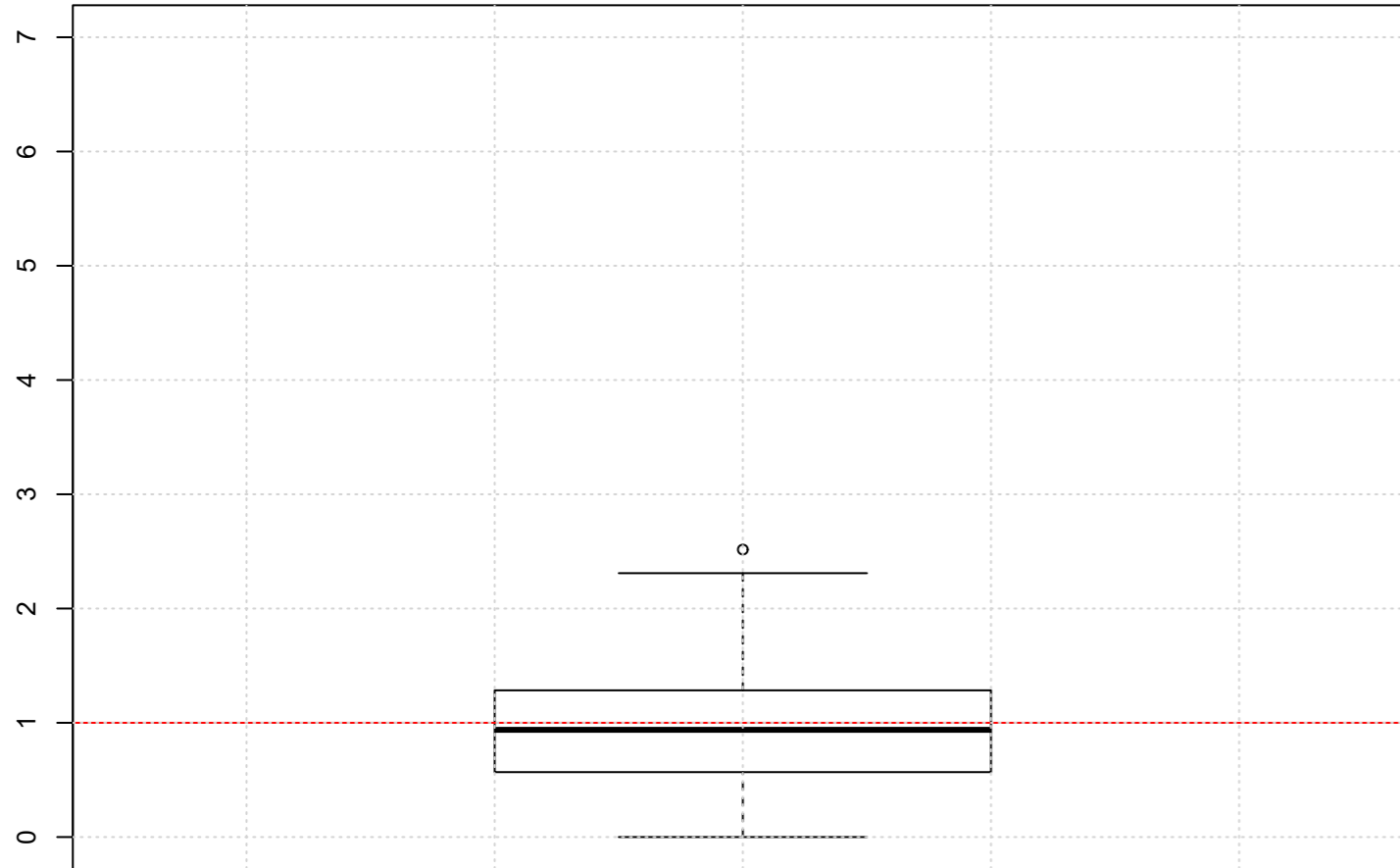

$\alpha=0.5$

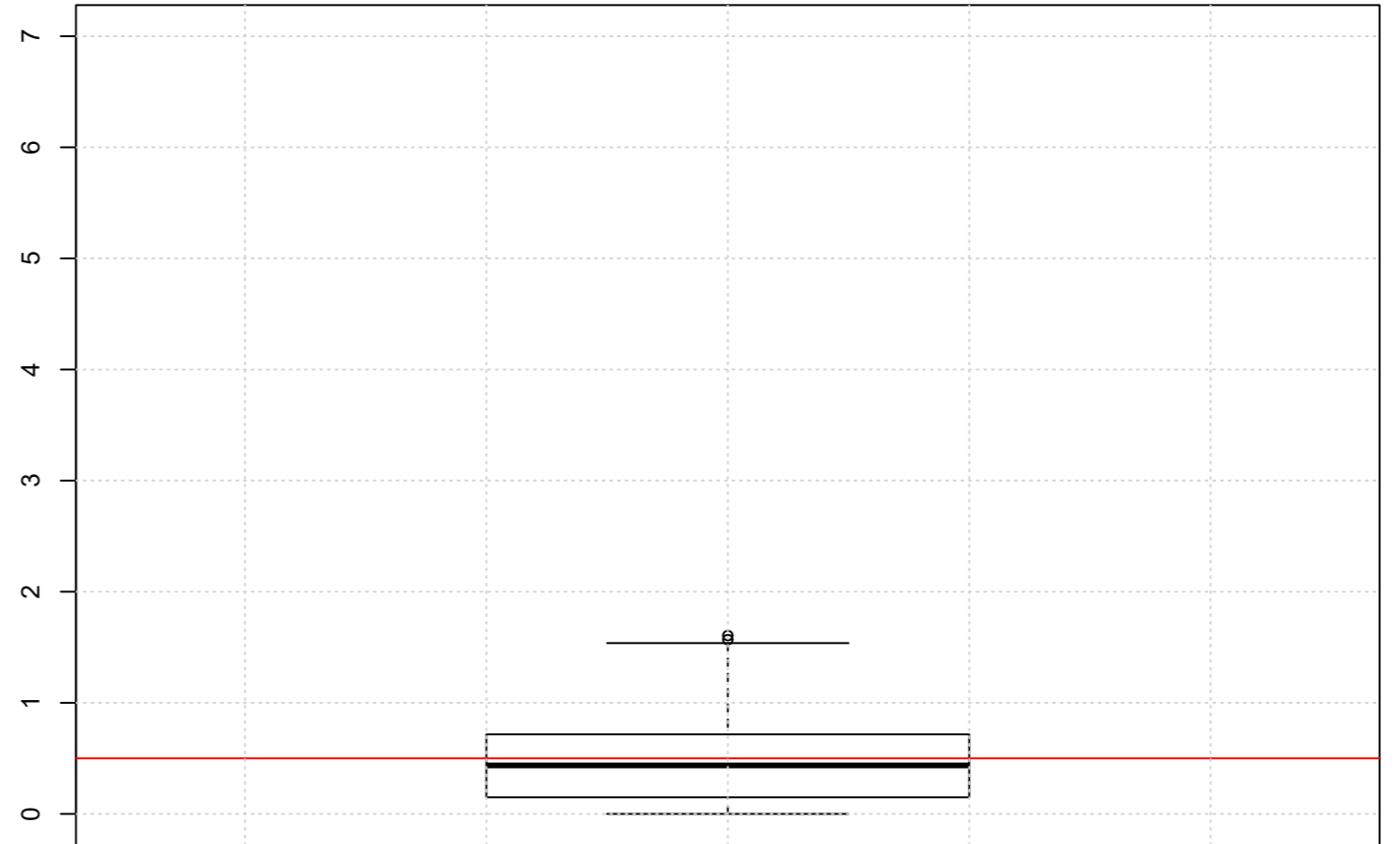

$\alpha=2$

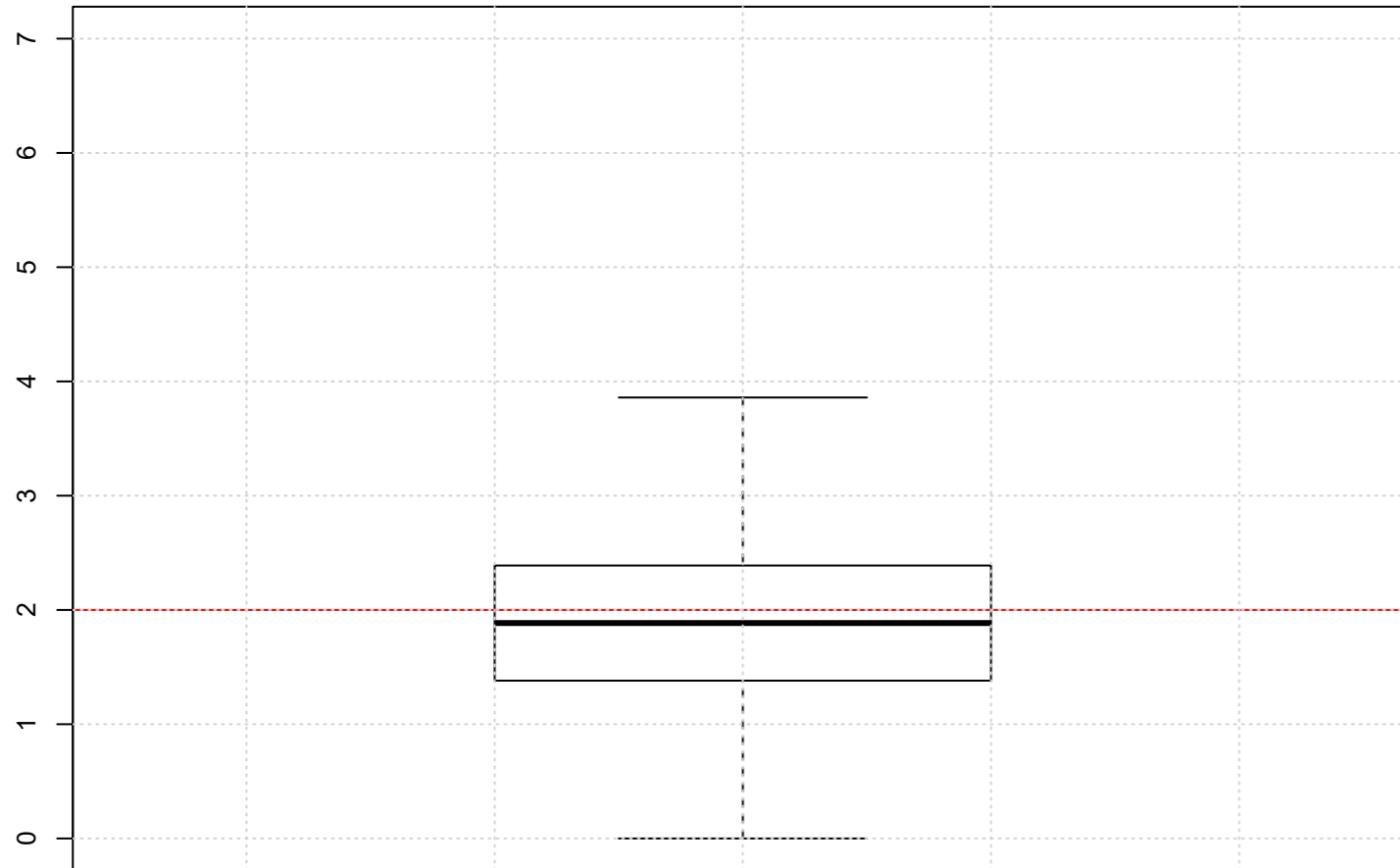

$\alpha=4$

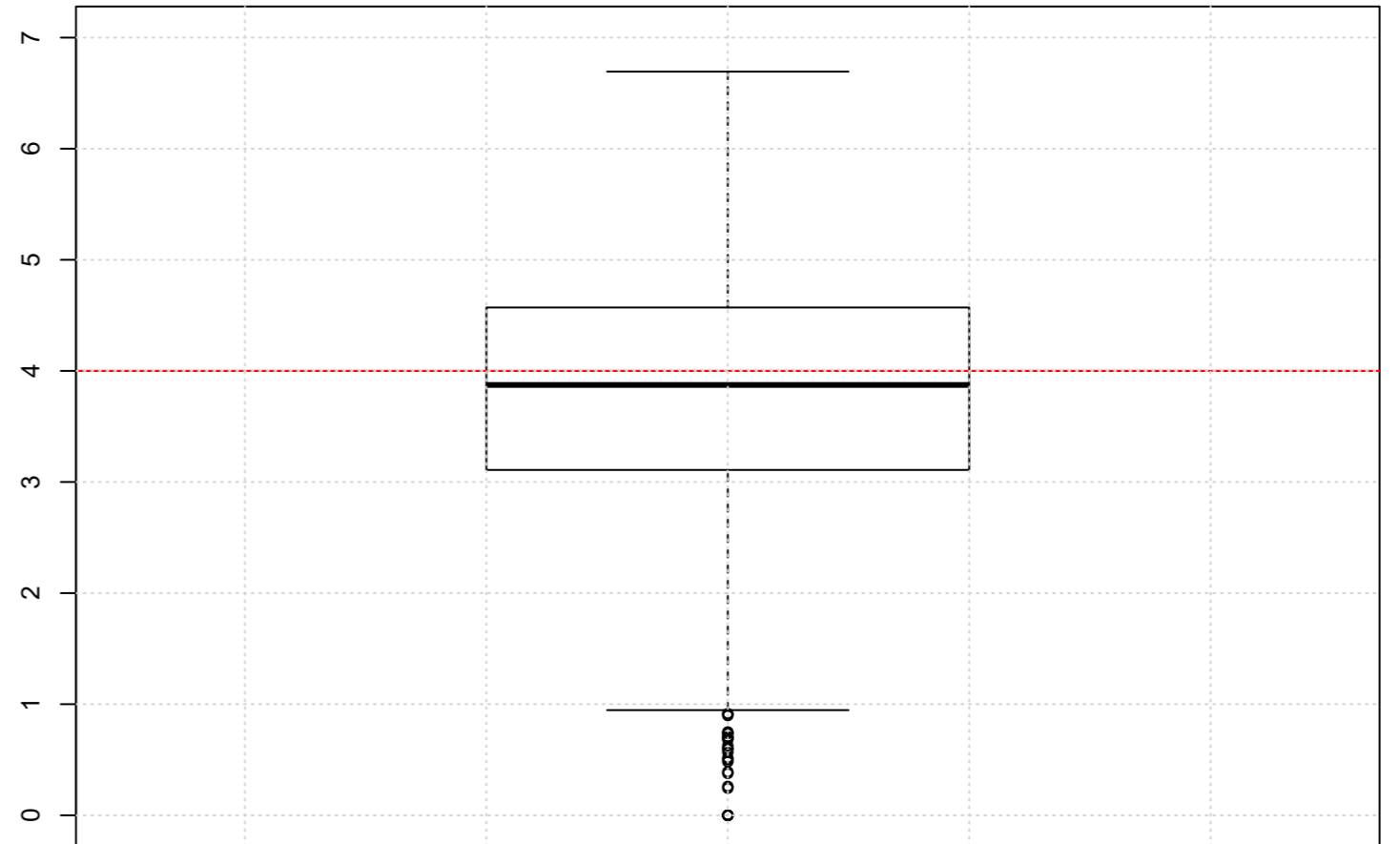

Supplement: Supplementary file 3 — Boxplots of the selected effect estimated with the RBS model in the simulation study. (PDF 17 kb) [file 12874_2019_747_MOESM3_ESM.pdf]
